# Treating SARS-CoV-2 Omicron variant infection by molnupiravir for pandemic mitigation and living with the virus: a mathematical modeling study

**DOI:** 10.1038/s41598-023-32619-z

**Published:** 2023-04-04

**Authors:** Qinyue Zheng, Chunbing Bao, Yunpeng Ji, Pengfei Li, Zhongren Ma, Xinwei Wang, Qingchun Meng, Qiuwei Pan

**Affiliations:** 1 School of International Affairs and Public Administration, Ocean University of China, Qingdao, China; 2 School of Management, Shandong Key Laboratory of Social Supernetwork Computation and Decision Simulation, Shandong University, Jinan, China; 3 Department of Gastroenterology and Hepatology, Erasmus MC-University Medical Center, Rotterdam, The Netherlands; 4 Department of Genetics, Inner Mongolian Maternal and Child Care Hospital, Hohhot, Inner Mongolian China; 5 Key Laboratory of Biotechnology and Bioengineering of State Ethnic Affairs Commission, Biomedical Research Center, Northwest Minzu University, Lanzhou, China; 6 Department of Engineering Mechanics, State Key Laboratory of Structural Analysis for Industrial, Equipment, Dalian University of Technology, Dalian, China

**Keywords:** Microbiology, Medical research

## Abstract

Treating severe COVID-19 patients and controlling the spread of SARS-CoV-2 are concurrently important in mitigating the pandemic. Classically, antiviral drugs are primarily developed for treating hospitalized COVID-19 patients with severe diseases to reduce morbidity and/or mortality, which have limited effects on limiting pandemic spread. In this study, we simulated the expanded applications of oral antiviral drugs such as molnupiravir to mitigate the pandemic by treating nonhospitalized COVID-19 cases. We developed a compartmental mathematical model to simulate the effects of molnupiravir treatment assuming various scenarios in the Omicron variant dominated settings in Denmark, the United Kingdom and Germany. We found that treating nonhospitalized cases can limit Omicron spread. This indirectly reduces the burden of hospitalization and patient death. The effectiveness of this approach depends on the intrinsic nature of the antiviral drug and the strategies of implementation. Hypothetically, if resuming pre-pandemic social contact pattern, extensive application of molnupiravir treatment would dramatically (but not completely) mitigate the COVID-19 burden, and thus there remains lifetime cost of living with the virus.

## Introduction

The emergence of the SARS-CoV-2 Omicron variant in November 2021 has caused new catastrophic waves across the globe including many European countries. This variant harbors a large number of mutations with more than 20 vital mutations in the spike protein alone. This has enabled the Omicron variant to escape from the existing COVID-19 vaccines and antibody therapies^1^. Furthermore, new subvariants of Omicron continuously emerge^2^. For example, the sub-lineage BA.2 has rapidly displaced the sub-lineages BA.1 and BA.1.1, but BA.2 has an increased transmission rate and decreased response to vaccine^3^.

Non-pharmaceutical interventions (NPIs), aiming at restricting social contacts, have been widely implemented across the globe albeit at different levels of intensity^4^. NPIs include within-population quarantine, school closure, working from home, takeaway and food delivery services, face mask wearing and social distancing. However, these measures have deteriorated consequences on society functions and economic growth. Although mandatory restrictions by government have been gradually lifted in most European countries, social activities in fact have not been fully resumed to the pre-pandemic patterns at the time of conducting this study. Based on the current population with pre-existing immunity level obtained by vaccination or recovery from SARS-CoV-2 infection, how social activities can be resumed is a top priority for policy making. While Omicron remains widely circulating, a rollback of confinement measures will unavoidably lead to an increase in new cases of infection. Thus, it is essential to prepare alternative strategies for mitigating the clinical burden, while living with the virus, as SARS-CoV-2 is inevitably becoming endemic^5^.

Classically, antiviral drugs are primarily developed for treating hospitalized COVID-19 patients with severe diseases to reduce morbidity and/or mortality, which are not intended for controlling pandemic spread. In fact, the nonhospitalized COVID-19 cases with no or mild symptoms largely contribute to virus spread. We postulate therapeutics that are capable of potently inhibiting SARS-CoV-2 could be explored for controlling pandemic spread, when implemented with specific strategies especially targeting the nonhospitalized population. Molnupiravir, an oral and safe antiviral drug approved for treating COVID-19, is a potential candidate in this respect^6^. A phase 2a clinical trial has shown that molnupiravir can effectively eliminate SARS-CoV-2, and the infectious virus was only detected in 1.9% of the treated group compared with 16.7% of the placebo group on day 3 post-treatment^7^. Based on the large phase 3 randomized trial enrolled 1433 patients, COVID-19–related hospitalizations or deaths accounted for 6.3% in the molnupiravir group and 9.2% in the placebo group^6^. The risk for COVID-19 severe illness was 11.4% with molnupiravir treatment compared to 15.2% with placebo, and the risk of death that was reduced by 89% (1.3% vs 0.1%) with molnupiravir^6^.

Although these clinical trials of molnupiravir are based on treating the ancestral and previous variants of SARS-CoV-2, our recent experimental study has demonstrated that it is also comparably effective against Omicron^8^. We hypothesize that potent inhibition of SARS-CoV-2 and shortened recovery time by molnupiravir treatment has a potential to limit virus spread in community. In this study, we aim to evaluate this non-canonical hypothesis by mathematical modeling. We estimated the impact of molnupiravir treatment on the Omicron-dominated pandemic waves by targeting different populations with various implementation strategies, taking three European countries—Denmark, the United Kingdom (UK), and Germany—as examples.

## Results

### Building a compartmental model for recapitulating SARS-CoV-2 transmission and assessing implementation strategies of molnupiravir treatment

**Figure 1:**
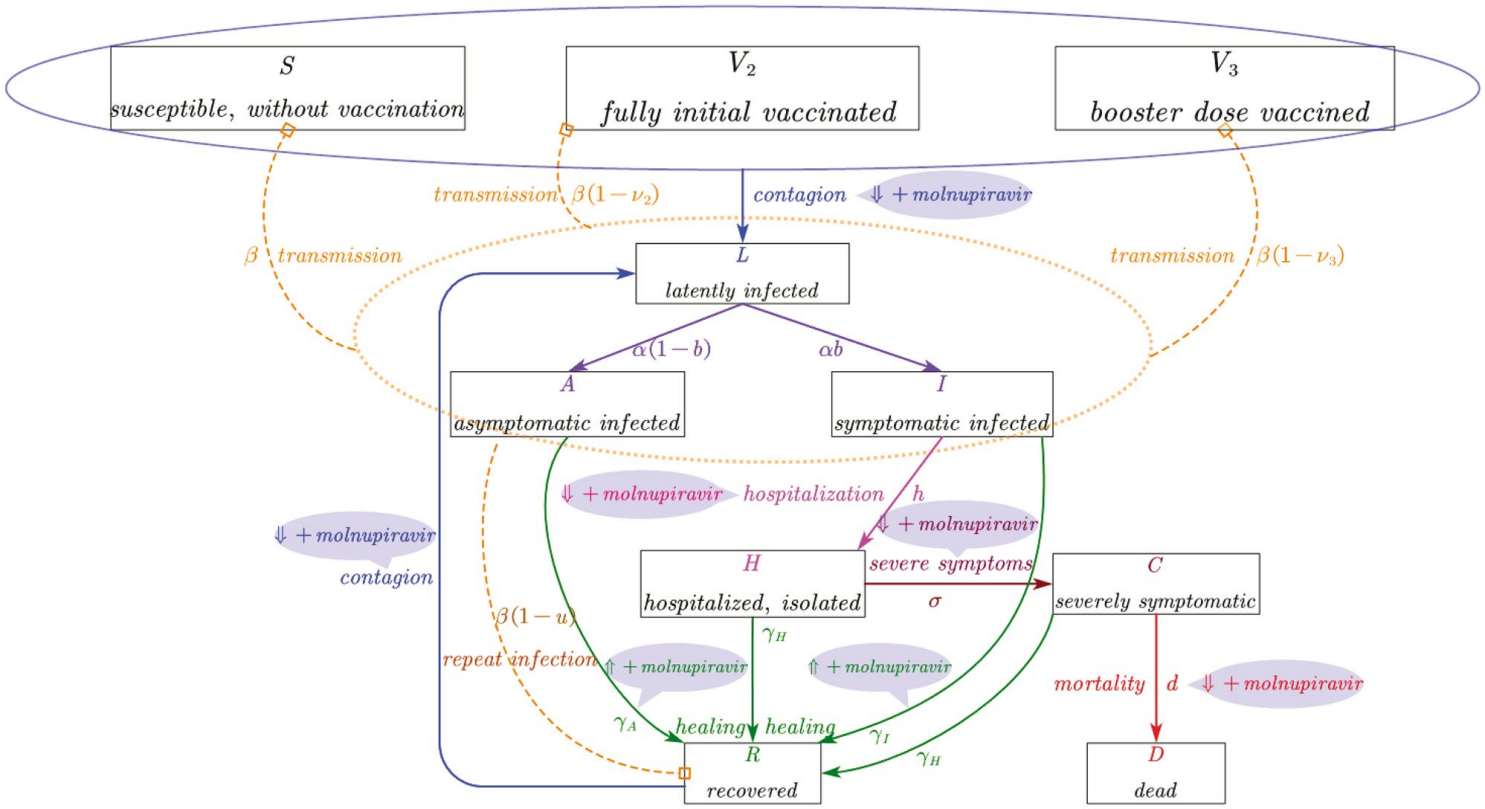
Schematic representation of the epidemiological model. In the mathematical compartment model: S, susceptible (uninfected, unvaccinated); V_2_, fully initial vaccinated (uninfected); V_3_, booster dose vaccinated (uninfected); L, latent (no symptom, with infectivity); A, asymptomatic carriers (infected, no symptom, with infectivity); I, symptomatic patients (actively infected with symptom, with infectivity); H, hospitalized (actively infected with symptoms, quarantined); C, Severe symptomatic (actively infected with severe symptoms, life-threatening, hospitalized, quarantined); R, recovered (healed, might be reinfected); D, dead (extinct). In this model, asymptomatic carriers mean asymptomatic infections who never develop symptoms but not pre-symptomatic infections who eventually develop into symptomatic infections.

We developed a mathematical model of SARS-CoV-2 transmission with ten compartments as shown in Fig. 1 (susceptible-fully initial vaccinated-booster dose vaccinated-latently infected-asymptomatically infected-actively infected-hospitalized-severely symptomatic-recovered-dead). The infections were stratified according to the characteristics of symptoms. This model considers protective immunity from vaccination and recovery after pervious infection. It is assumed that COVID-19 vaccine-induced protection against Omicron variant wanes with time, and the protection against reinfection is partial. Our model considers heterogeneous contacts as occurring in real-world, namely, households and non-households. The level of social contacts is expressed in accordance with the traced human encounters and social mixing patterns between households and non-households. In order to accurately capture the epidemic spread, we adopted parameters from widely referred studies and authentic sources (Supplement Information). Our model simulation in the baseline scenario without molnupiravir treatment fitted well with the real-world reported cases of three European countries (Denmark, UK, and Germany) in April and May, 2022 (Fig. S1).

We simulated the COVID-19 burden under different levels of social activity in the coming two months from April 01, 2022, when the Omicron BA.2 variant dominated in Denmark, UK and Germany. We assume that the average infectivity would be reduced by 46.4% corresponding to accelerated viral RNA clearance and infectious virus elimination after receiving 800 mg molnupiravir, and the risk for symptom diseases would be reduced by 44.6%^6,7^. We incorporated these key effects to assess the dynamic impact of implementing oral molnupiravir treatment using various strategies on limiting SRAR-CoV-2 spread and mitigating clinical burden in these three representative countries. The settings and parameters of our model and implementation strategies of molnupiravir treatment are detailed in the Methods Section and Supplementary Information.

### Recapitulating the transmission dynamics and simulating pandemic burden of Omicron waves

**Figure 2:**
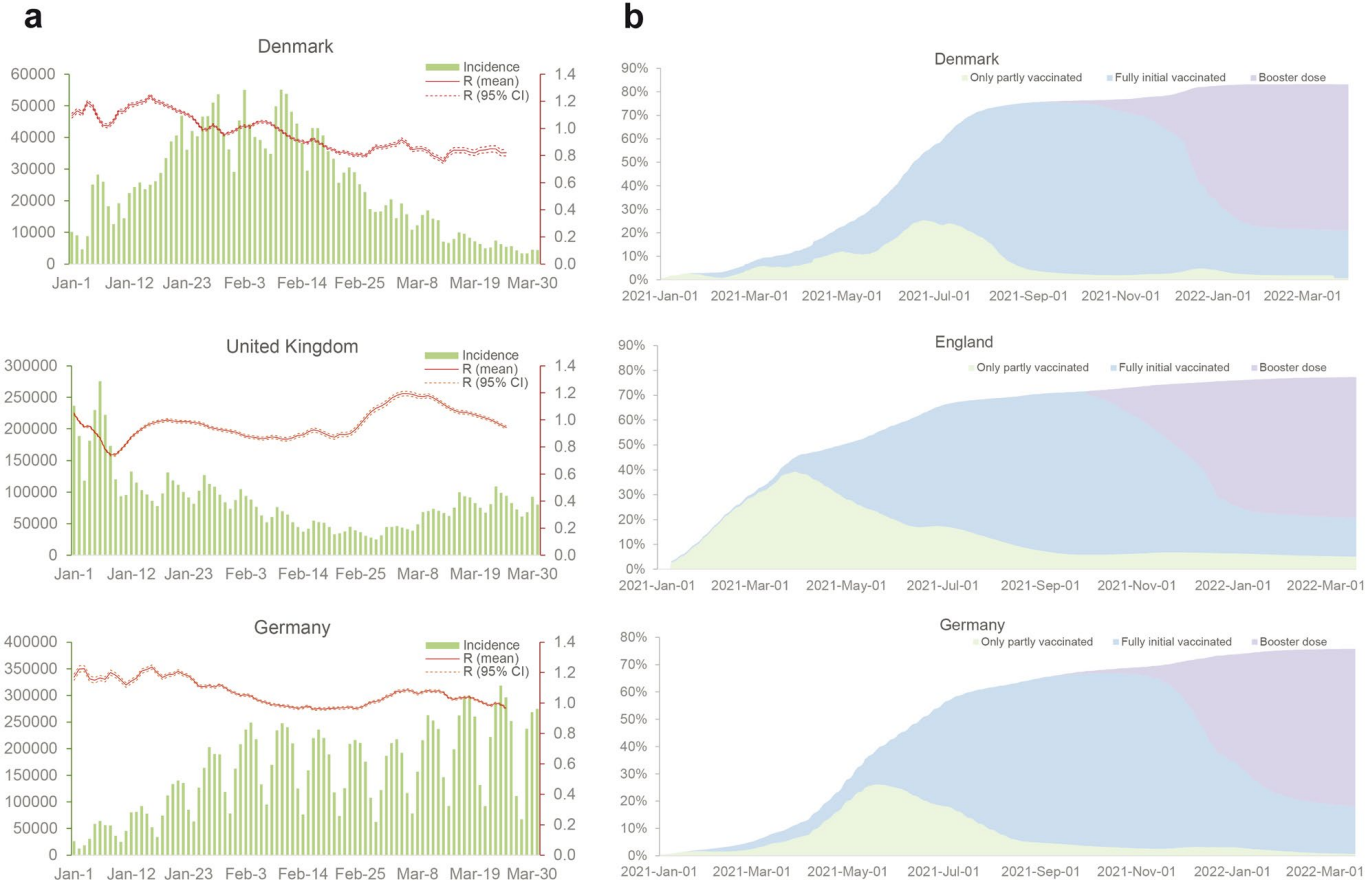
Simulating the transmission dynamics and pandemic burden of Omicron waves. (**a**) The time-varying reproduction number estimation and epidemiological dynamics. (**b**) The dynamics of vaccination coverage by types. Incidence curve represents the number of daily new confirmed cases reported by WHO. The time-varying reproduction number was computed from epidemic incidence curves recorded by the WHO, and the dotted lines parts represent the 95% confidence interval. The vaccination data is retrieved from Our World in data.

The reproduction number ($\Re_{{\text{t}}}$) is the average secondary cases generated by per infected individual, showing how fast the virus is spreading. The $\Re_{{\text{t}}}$ value depends on multiple factors including the epidemiological features of the SARS-CoV-2 variant, the population vaccination status and implementation of social NPIs. In order to recapitulate the evolving transmission dynamics, we estimated the near-real-time $\Re_{{\text{t}}}$ values for Denmark, UK and Germany (Fig. 2a). The $\Re_{{\text{t}}}$ value of Denmark was lower than 1 after February 10, 2022, and maintained around 0.85 in March. In UK, $\Re_{{\text{t}}}$ was less than 1 before February 24, but hovering around 1.1 in March. In Germany, $\Re_{{\text{t}}}$ was over 1.1 in January, and fluctuated around 1 in February and March (Fig. 2a). Given the progress and coverage of vaccination implementation are similar in these three countries (Fig. 2b), we postulate that their distinct transmission dynamics may be attributed to multiple factors including the different levels of NPIs and social contacts.

**Figure 3:**
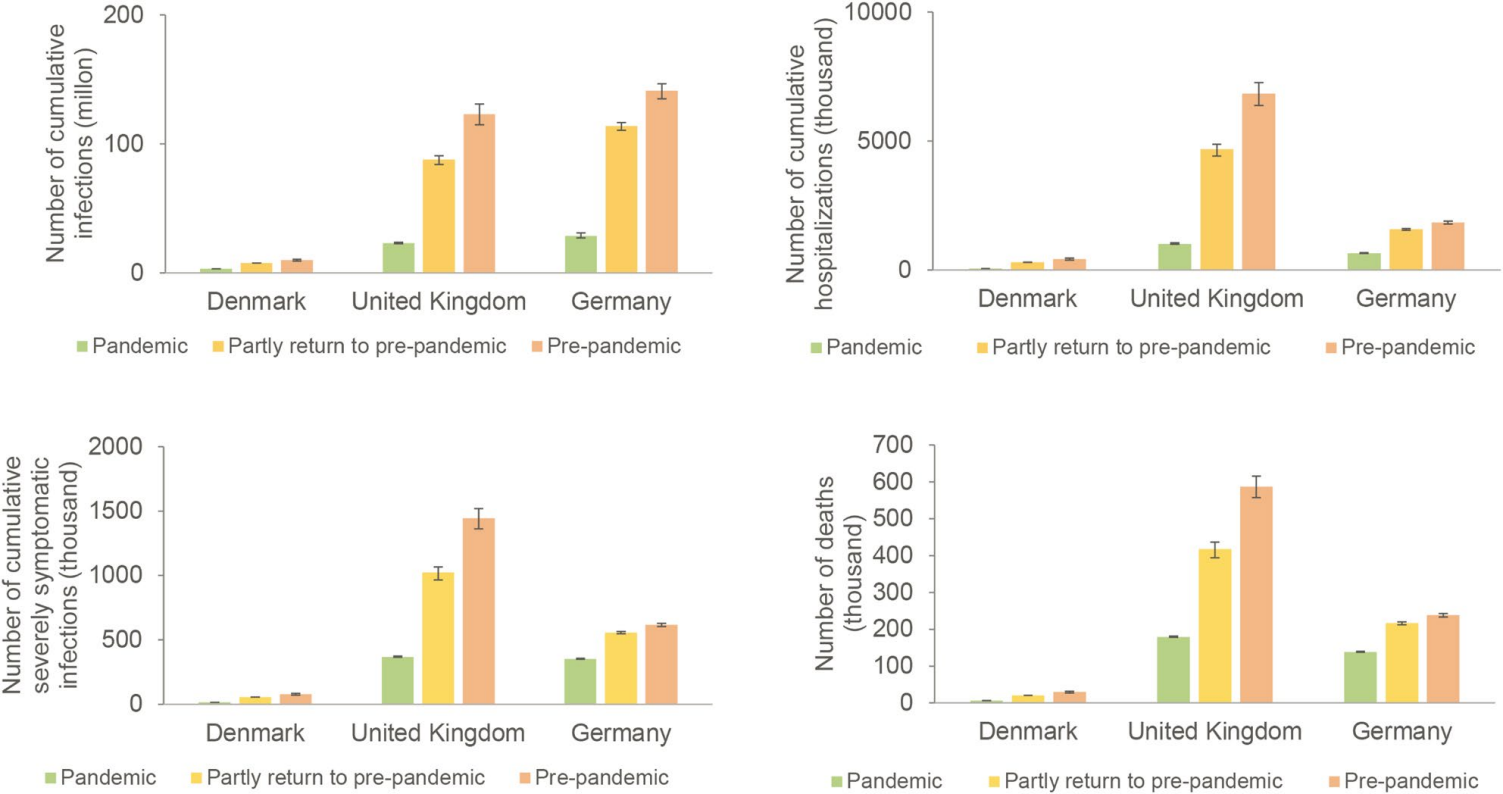
Burden of COVID-19 under different levels of social activity. The simulated cumulative number of infections includes both asymptomatic carriers and symptomatic patients. The level of social activity is expressed in terms of social contacts, which are estimated according to the average number of human social contacts to a person and assuming a homologous secondary attack rate. The pandemic social activities scenario matched the epidemiological characterization in Week 12, 2022 (the reproduction number is around 0.9). The scenario of partially returning to pre-pandemic social activities refers to the number of close contacts reported in technical briefing of SARS-CoV-2 variants identified by UK Health Security Agency from January to February in 2022 (the reproduction number is around 1.5). The scenario of resuming pre-pandemic social activities is set in accordance with mixing patterns in social networks before the COVID-19 pandemic. (*Source* https://doi.org/10.1016/j.socnet.2007.04.005). The Error lines represent the 95% confidence interval related to parameters.

Therefore, we simulated the impact of three scenarios of social activity levels which recapitulate the pandemic, partially resuming to pre-pandemic and fully resuming pre-pandemic social contact patterns, respectively. We projected the coming two months from April 01, 2022 in our model. We found profound impact of social contact patterns on COVID-19 burden (Fig. 3). If the current social contact patterns would be maintained, the cumulative new infections would be 69.1 (95% CI 56.5–88.4) thousand in Denmark, 1.9 (95% CI 1.5–2.7) million in UK, and 7.5 (95% CI 5.9–9.7) million in Germany from April 01 to May 31. The cumulative new deaths would be 602 (95% CI 559–663) in Denmark, 11.3 (95% CI 10.0–13.3) thousand in UK, and 9.3 (95% CI 8.4–10.7) thousand in Germany. These estimates correspond to 99.0% (95% CI 98.8–99.1%), 98.1% (95% CI 97.6–98.4%), and 93.8% (95% CI 92.3–94.8%) reduction of infections, and 97.5% (95% CI 97.3–97.6%), 97.3% (95% CI 97.0–97.4%), and 91.4% (95% CI 90.5–92.0%) reduction of deaths comparing to the scenario of resuming pre-pandemic social activity level in Denmark, UK, Germany, respectively. Similar results were observed on the burden of hospitalization, and severely symptomatic infections (Fig. 3). Even partial resumption of pre-pandemic level, for example in Denmark, there would be 4.5 (95%CI 4.4–4.5) million infections, 41.9 (95%CI 41.1–42.3) thousand severe cases and 14.8 (95%CI 14.4–15.0) thousand deaths (Fig. 3).

### Estimating the impact of unconventional applications of molnupiravir treatment for pandemic mitigation

**Figure 4:**
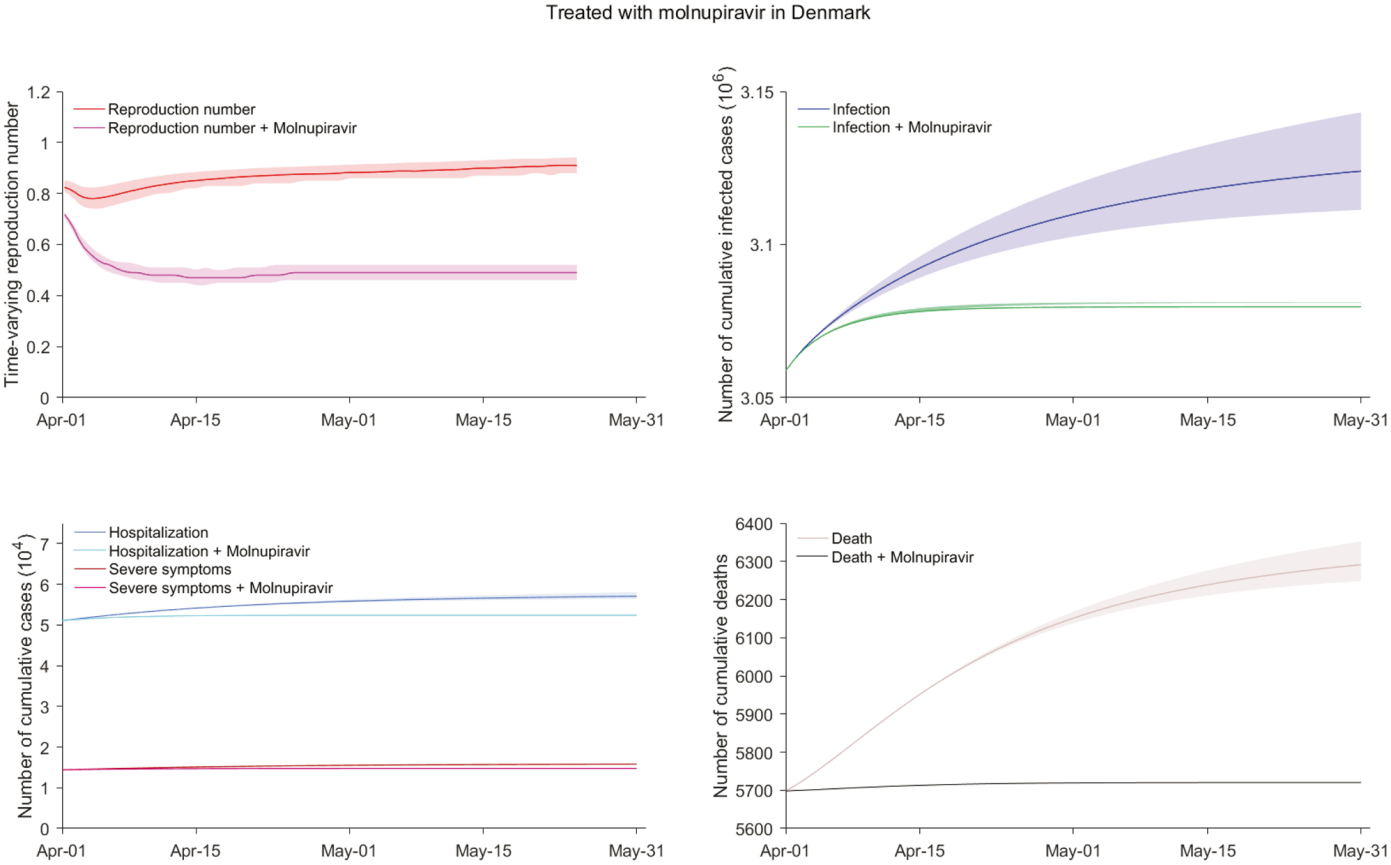
The impact of molnupiravir treatment for all nonhospitalized cases in Denmark. All nonhospitalized cases including latent ones, asymptomatic carriers, and active patients are treated with molnupiravir. The social activity level follows the situations in Week 12, 2022 in the country. The dominating lineages in Denmark (98.8% BA.2 and 1.2%BA.1) are referred from the national integrated surveillance system. The protection against infection of vaccine booster dose is assumed to decrease over time. The protection against reinfection is assumed as 9.3%. The implementation of molnupiravir treatment is assumed since the beginning of the simulation (01 April, 2022). We assumed that the infected cases in our models have not been previously treated with molnupiravir. Cases receiving molnupiravir treatment from day 1 after infection. Molnupiravir treatment was assumed to reduce the transmission rate, and mitigate risk of severe symptoms, hospitalization and death. The shadow parts represent the 95% confidence interval related to parameters.

For mitigating COVID-19 pandemic, we believe that the use of antiviral therapy should go beyond hospitalized cases, as these patients are isolated with limited contribution to virus spread. Assuming the entire nonhospitalized population of COVID-19 including latent, asymptomatic and symptomatic cases could be treated with molnupiravir, there would be a profound reduction by 68.0% (95% CI 62.7–73.5%) in Denmark (Fig. 4), 76.0% (95% CI 70.6–81.4%) in UK (Fig. S2), 80.3% (95% CI 75.8–83.4%) in Germany (Fig. S3) in the total number of infections in April and May, 2022. $\Re_{{\text{t}}}$ would be stabilized at 0.5 for Denmark, 0.6 for UK, and 0.7 for Germany by the end of May if treated with molnupiravir.

**Figure 5:**
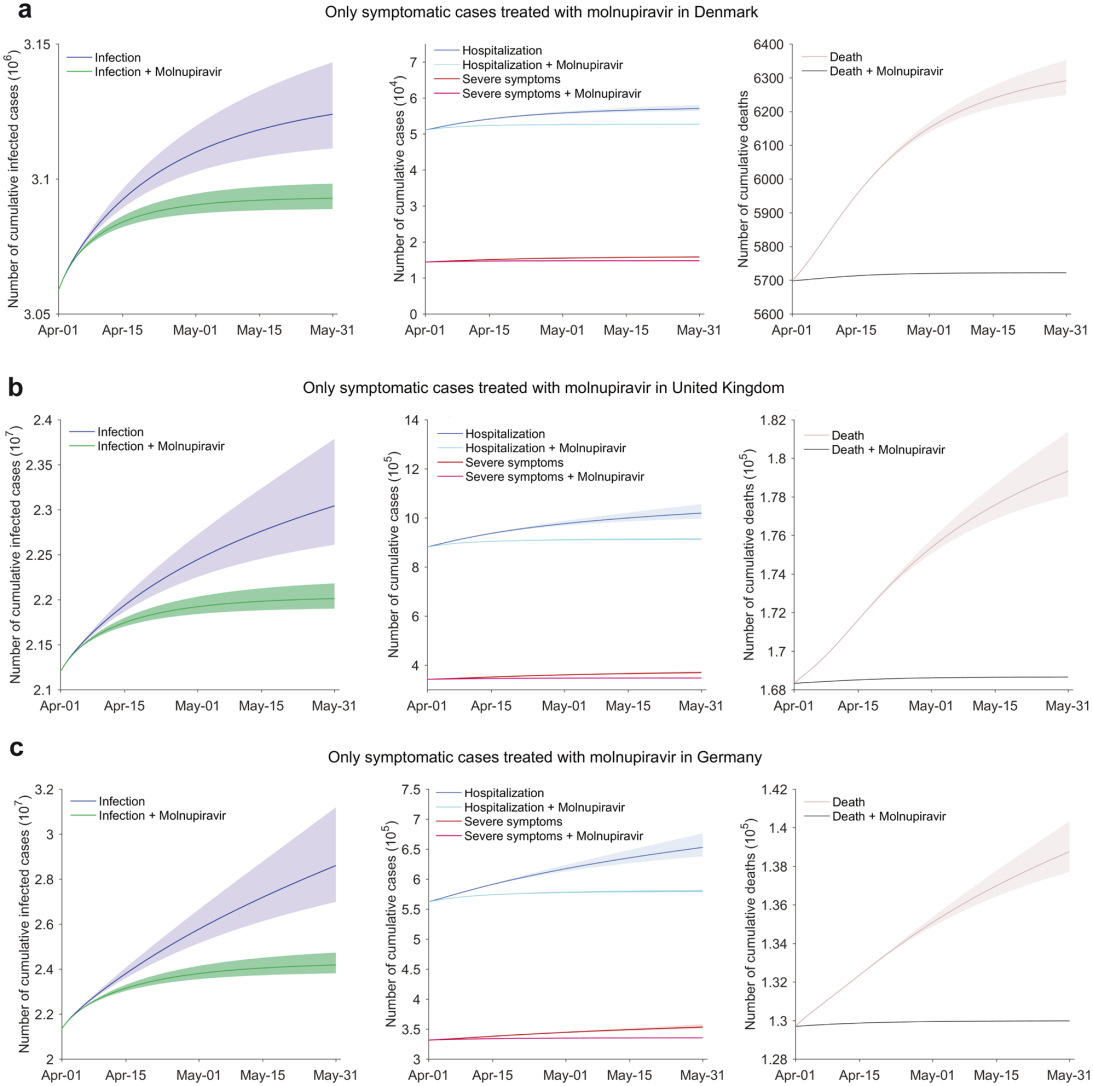
The impact of molnupiravir treatment for nonhospitalized patients with symptoms. (**a**) Denmark. (**b**) UK. (**c**) Germany. Treatment with molnupiravir after the onset of symptoms in nonhospitalized cases. The settings of social activity level, the reinfection proportion, dominating pango lineages, the treatment implementation time, and the molnupiravir treatment effectiveness for individuals are identical to those in Fig. 4. The shadow parts represent the 95% confidence interval related to parameters.

In real-world, asymptomatic carriers are usually unaware of the infection, and therefore it is difficult to prescribe antiviral treatment to them. We thus simulated the application of molnupiravir to symptomatic patients only. This would already result in a reduction of infections by 47.7% (95% CI 42.8–53.2%) in Denmark (Fig. 5a), 56.0% (95% CI 50.4–62.1%) in UK (Fig. 5b) and 60.7% (95% CI 57.3–64.3%) in Germany (Fig. 5c), thus preventing 31.1 (95% CI 22.5–44.9) thousand people from infection and 1.0 (95% CI 0.9–1.2) thousand from severe symptoms in Denmark (Fig. 5a), preventing 1.0 (95% CI 0.7–1.6) million infections and 22.2 (95% CI 18.8–27.8) thousand severe cases in UK (Fig. 5b), and preventing 4.4 (95% CI 3.3–6.1) million infections and 17.9 (95% CI 15.4–21.6) thousand severe cases in Germany (Fig. 5c), respectively.

The effectiveness of molnupiravir treatment on pandemic mitigation appears to be positively correlated with the transmission dynamics in these three countries. Similarly, we found that molnupiravir treatment is more effective in containing the infections of Omicron XE with greater transmissibility (82.0% reduction) comparing with BA.1 (61.3% reduction) and BA.2 (76.9% reduction) in the setting of UK (Fig. S4). Furthermore, Omicron is highly sophisticated in escaping from the existing vaccines, resulting in high rate of reinfection^9^. We thus also modeled scenarios of molnupiravir treatment assuming different rates of reinfection (Fig. S5).

### Simulating the impact of molnupiravir treatment coverage and timeliness

**Figure 6:**
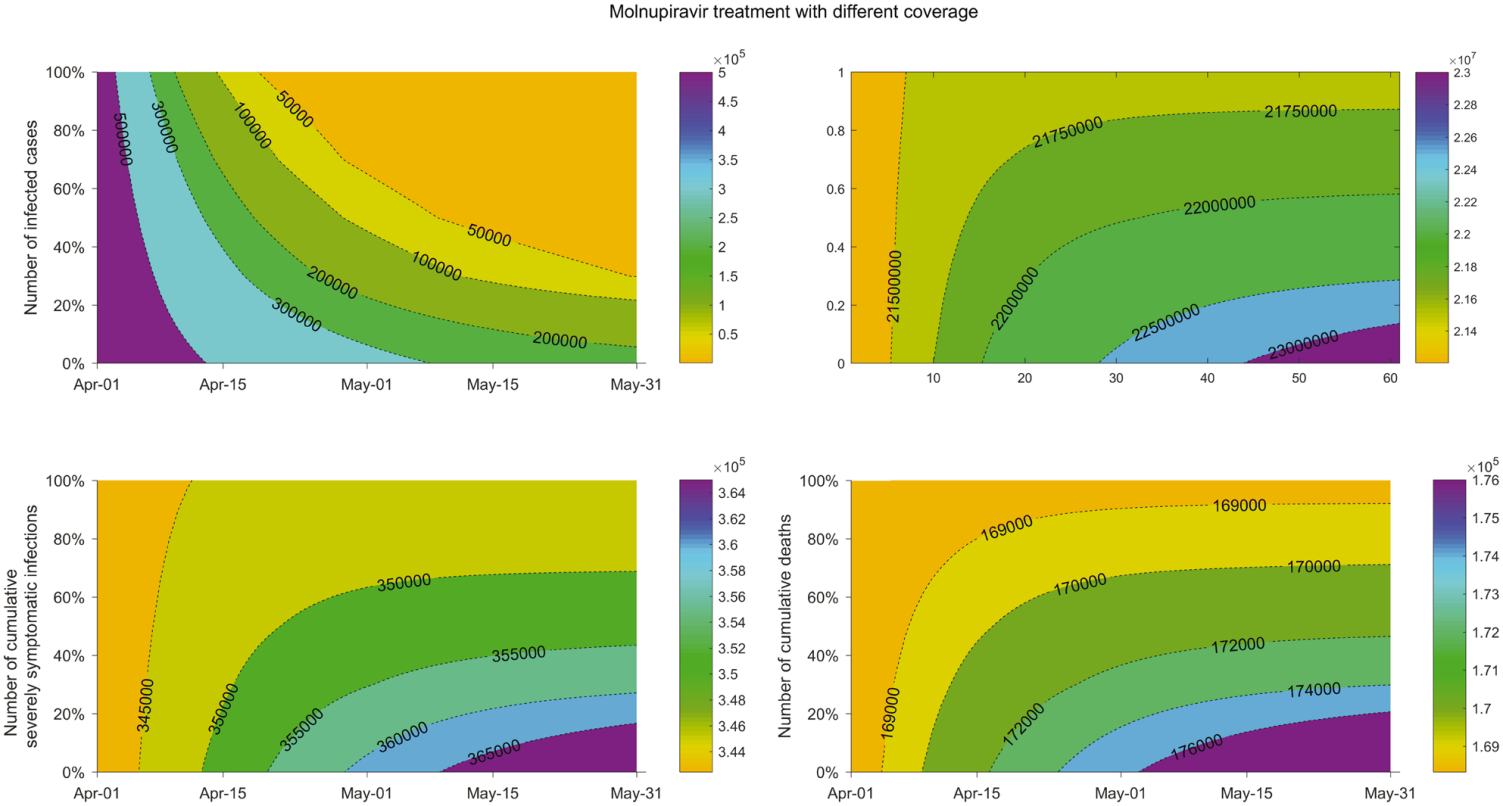
The impact of different coverages of molnupiravir treatment in the setting of UK. In this scenario, nonhospitalized cases including latent ones, asymptomatic cases, and symptomatic patients would be proportionally treated with molnupiravir. The settings of social activity level, the reinfection proportion, dominating pango lineages, and the molnupiravir treatment effectiveness for individuals are identical to those in Fig. 4. Contours represent epidemic performance under different coverage and time.

Considering the issues of availability and affordability in real-world, we modeled different coverages of molnupiravir use for COVID-19 cases in the setting of UK, which showed clear dose-dependent effects (Fig. 6). In these scenarios, all nonhospitalized infections including latent ones, asymptomatic carriers, actively infected patients were proportionally treated with molnupiravir. When only treating 30% of the cases, there would be a total reduction of new infections by 44.5% in the next two months, with dramatic reduction of severely symptomatic patients (47.2%) and deaths (53.7%). When the coverage reaches 50% and 70%, the cumulative new cases would be reduced by 73.2% and 85.8%, and the reduction of cumulative deaths would be 8.9 thousand and 10.4 thousand, respectively.

**Figure 7:**
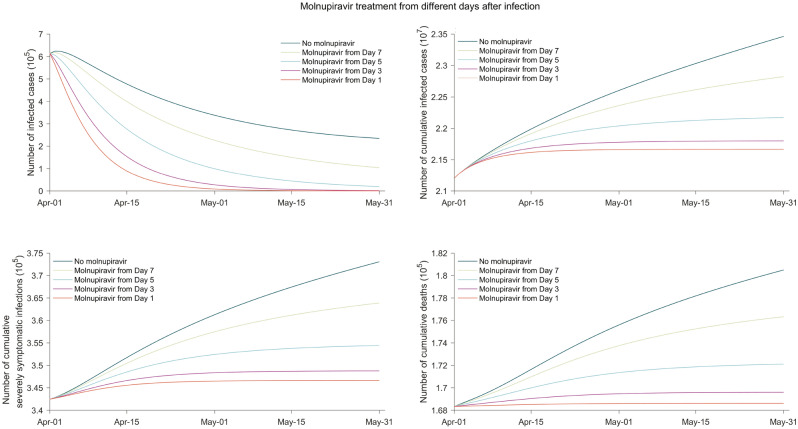
The impact of the timeliness of applying molnupiravir treatment in the setting of UK. In this scenario, all nonhospitalized cases are proportionally treated with molnupiravir. The timeliness is referring to molnupiravir treatment starting on which day post-infection. The settings of social activity level, the reinfection proportion, dominating pango lineages, and the molnupiravir treatment effectiveness for individuals are identical to those in Fig. 4.

We further estimated the effects of timeliness (e.g., dosing 1, 3, 5, and 7 days after infection) (Fig. 7) in receiving molnupiravir in the nonhospitalized population, including latently infected, asymptomatic carriers, actively infected patients. When applying molnupiravir treatment since day 3 after infection, substantial reduction in total numbers of new infection (73.6%), hospitalization (77.6%), severely symptomatic cases (79.4%) and death (89.6%) throughout two months were observed. Similar effects, although to a less extent, could be achieved in the scenarios of molnupiravir treatment from day 5 and 7 after infection, where the cumulative numbers of new infections would be reduced by 57.1% and 28.4% and the cumulative numbers of deaths would be reduced by 69.0% and 34.2%, respectively.

### Prospective for returning to pre-pandemic social activities under universal application of molnupiravir treatment

Although immunity from natural infection and vaccination is building up in the global population, the feasibility of absolutely resuming pre-pandemic social contacts and living with the virus remains ambiguous. We envisioned this scenario assuming extensive application of oral molnupiravir treatment.

**Figure 8:**
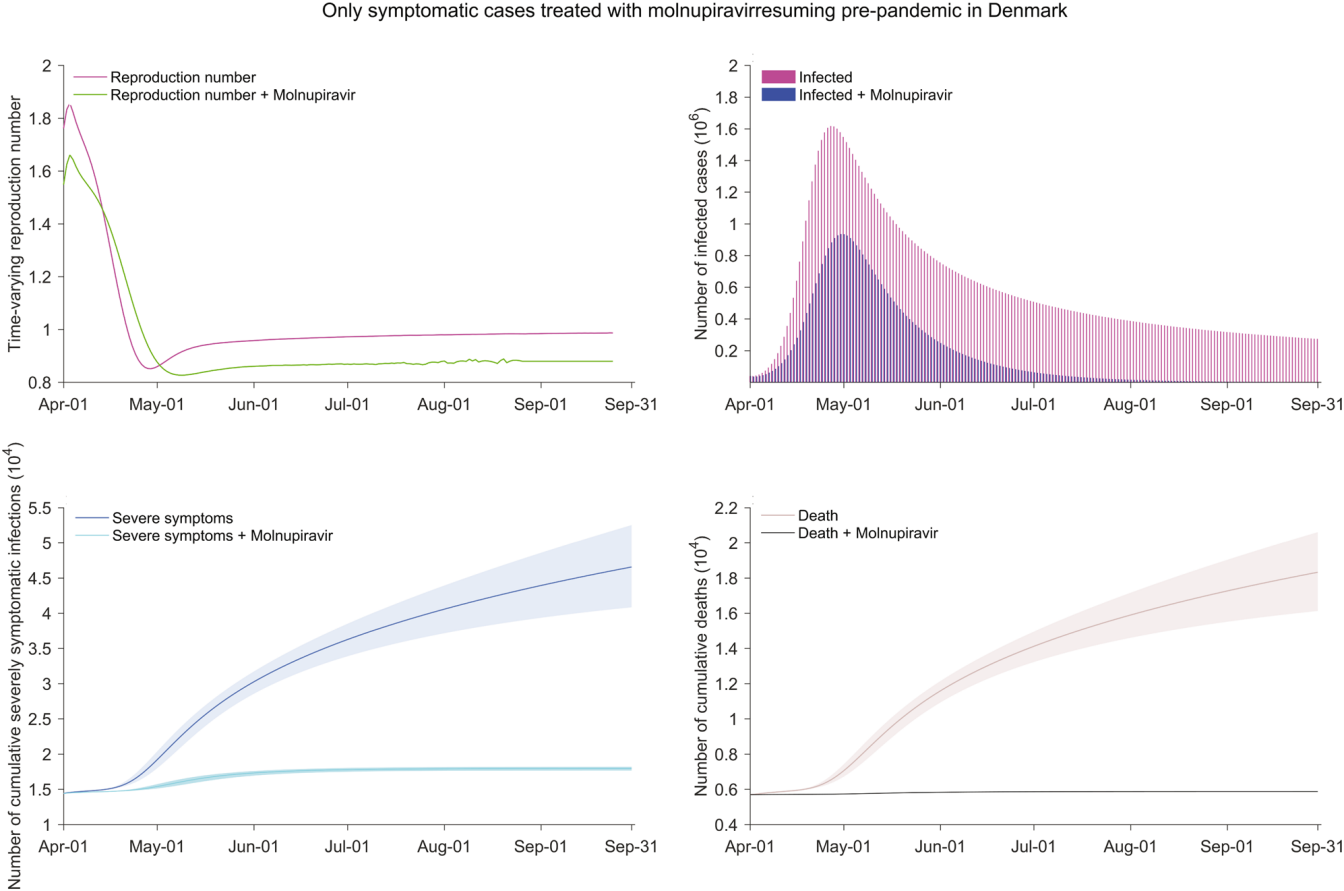
The impact of molnupiravir treating nonhospitalized patients with symptoms resuming pre-pandemic social activities in Denmark. Treatment with molnupiravir after the onset of signs or symptoms in nonhospitalized cases. The social activity level is in accordance with mixing patterns in social networks before the COVID-19 pandemic. The number of infected cases represents the current situation of existing infected ones including asymptomatic carriers and active patients. The settings of the reinfection proportion, dominating pango lineages, the treatment implementation time, and the molnupiravir treatment effectiveness for individuals are identical to those in Fig. 4. The shadow parts represent the 95% confidence interval related to parameters.

Assuming returning to pre-pandemic social activity pattern in Denmark, $\Re_{{\text{t}}}$ would be maintained around 0.99 in the coming 6 months (by 31 September, 2022). The total numbers of infections, severe cases and deaths would be substantial. Implementation of molnupiravir treatment for symptomatically infected cases is estimated to reduce total infections by 54.8% (95% CI 49.0–59.4%), severe cases by 89.0% (95% CI 87.7–90.1%) and deaths by 98.6% (95% CI 98.4–98.7%). However, there would still be 6.9 million infections, 3.5 thousand severe cases and 176 deaths occurring during the coming 6-month period (Fig. 8). Similar results are observed in the settings of UK (Fig. S6) and Germany (Fig. S7).

## Discussion

In this study, we modeled the potential of oral antiviral drugs such as molnupiravir for mitigating COVID-19 pandemic, taking the Omicron waves in three European countries as a demonstration. Our mathematical modeling showed that treating COVID-19 cases who do not require hospitalization can limit SARS-CoV-2 spread and indirectly reduce the burden of hospitalization and patient death. The effectiveness of this approach on mitigating COVID-19 spread, healthcare burden and patient death depends on the intrinsic nature of the antiviral drug, the epidemic setting, the targeting population, the coverage, and the timeliness of applying the treatment.

Conceptually, it appears unnecessary to treat nonhospitalized cases, as they are quarantined at home and will recover spontaneously. In worse case, they would only infect some family members. In reality, the levels of actually implemented control measures and the responses from the general public can vary dramatically among different countries and regions^10^. For example, Europe has encountered serious pandemic fatigue with great concerns of governmental and public commitment to COVID-19 prevention measures. This has triggered a rapid surge of the Omicron-dominant wave across different European countries^11^. Community transmission driven by asymptomatic careers and cases with mild symptoms sustained most of the COVID-19 waves^12^. As testing has been scaled up worldwide^13^, detecting pre-symptomatic and asymptomatic infections already becomes realistic at population level. We expect that the availability of an oral, safe, cheap and effective antiviral drug against SARS-CoV-2 could motivate COVID-19 testing and treatment for suspected cases with mild symptoms and asymptomatic careers who know their contact history with infected cases, which could indirectly but dramatically limit SARS-CoV-2 spread.

In this study, we focused on modeling molnupiravir, which was granted Emergency Use Authorization by the US Food and Drug Administration (FDA) in December 2021 for treating COVID-19 patients. Molnupiravir is a small-molecule nucleoside analog that targets viral RNA polymerase to directly inhibit viral replication^14^. In our study, the impact of molnupiravir in mitigating COVID-19 pandemic is largely dispensable of its efficacy in reducing mortality. It is mainly attributed to the dramatic reduction of infectious virus titters in treated nonhospitalized cases, since it is a potent direct-acting antiviral drug^7^. By treating these infected but nonhospitalized cases, molnupiravir limits the spread of SARS-CoV-2, and thereby indirectly reduces the hospitalization burden and mortality. One important feature of molnupiravir is an oral antiviral drug, which is feasible for massive prescription to nonhospitalized population. In this respect, Paxlovid, another oral antiviral therapy authorized for treating COVID-19^15^, also has potential to be explored for such application. In contrast, remdesivir, the first approved antiviral drug for treating COVID-19, is likely not suitable for treating nonhospitalized cases, because it has to be administered intravenously by a health care professional^16^.

At the time of performing this research, control measures have been gradually lifted in many European countries, but the level of social activities have not been fully resumed to the pre-pandemic era. This could be attributed to psychological^17^ and habitual factors, as well as voluntary face mask wearing and social distancing especially by vulnerable populations having knowledge and awareness of COVID-19^18^. Therefore, our scenario of resuming to pre-pandemic social activity is not equivalent to the real situations in Europe. Our estimated numbers in this scenario are expected to be higher than those reported from the real-world.

There are several other critical issues to be carefully assessed, when considering the feasibility and effectiveness of applying antiviral treatment to limit epidemic spread. These important factors include availability, accessibility and affordability of the antiviral drug. The U.S. government has paid around $700 per five-day course of molnupiravir, and $530 for a 5-day course of Paxlovid. These prices would be too high for treating large nonhopitalized populations as proposed in our study, but this high price issue can change in the near future^19^. Furthermore, the availability of these new COVID-19 drugs is expanding in low- and middle-income countries through agreement of generic drug licenses^20^. For example, a 5-day course of 40 capsules with the generic version of molnupiravir produced in India will cost less than $20^21^. In practice, global implementation of antiviral treatment for limiting epidemic spread is not always feasible even at low cost. However, specific settings, such as refugee camps, may be preferential targets. Because of high population density, fragile health system and poor access to vaccines, refugee camps have unique challenges in responding to epidemics^22^, whearas deploying oral antiviral therapy may serve as an effective countermeasure.

Large-scale implementation of antiviral monotherapy needs to also consider potency of the drug and timeframe of the application. Based on experience of antiviral monotherapy, the drug will place a selective pressure for resistance mutations, which may emerge and spread to the population overtime. Current data remain insufficient to ascertain how high the barrier of resistance is with SARS-CoV-2 to molnupiravir^23^. Combinational therapies are being increasingly explored for treating viral infections in the clinic. Combinational therapies often exhibit synergism, and can better prevent the development of drug-resistant strains. Therefore, antiviral drug combinations are a favorable option for front line therapy against poorly characterized emerging viruses and re-emerging drug-resistant viral variants^24^.

The concept of using antiviral treatment as an effective approach of epidemic control is not completely new. For example, early detection and applying antiretroviral therapy have been shown as an effective method to stop secondary transmission of HIV, and to control the HIV epidemic^25^. At the beginning of the 2009 influenza A H1N1 pandemic, the US government released 11 million courses of antiviral drugs from the stockpile as a potential countermeasure. A mathematical modeling has suggested that targeted antiviral prophylaxis has potential as an effective countermeasure for containing influenza until a sufficient quantity of vaccines become available^26^. Thus, the implications of deploying antiviral agents to combat epidemics go beyond the current COVID-19 pandemic^27^, and can serve as preparation for future epidemics, in particular the pandemic risk associated with highly pathogenic avian influenza viruses^28^.

Of note, there are some limitations of the study. First, we did not incorporate the key risk factors for developing severe COVID-19, such as age and comorbidities. Because this study mainly focuses on the nonhospitalized population and the spread of the epidemic, but not the clinical outcomes. Furthermore, we are not able to access these comprehensive demographic and clinical data for the three countries that we have modeled. Second, we assessed the impact in a relatively short timescale, because to accurately recapitulating a longer period of pandemic is technically very challenging with modeling. However, future studies should explore this possibility which would shed insight on the long-term impacts of this strategy. Finally, we employed molnupiravir as a “model” drug, but do not advocate the widespread use of molnupiravir per se to limit COVID-19 spread in real-world.

In summary, this modeling study provided proof-of-concept that treating nonhospitalized COVID-19 cases by molnupiravir could have an impact in limiting SARS-CoV-2 spread and mitigating the clinical burden of COVID-19. Importantly, this strategy could be generalized to many future medications that can directly inhibit SARS-CoV-2, even though they may not be sufficient to cure severe COVID-19 patients. Thus, our findings provided new strategies for effectively combating the ongoing COVID-19, as well as preparation for future epidemics and pandemics. Nevertheless, widespread use of antiviral treatment for mitigating epidemic spread requires in-depth investigation and careful assessment with multi-stakeholder involvement in particular the relevant regulatory bodies.

## Methods

### SARS-CoV-2 transmission model

We developed a model of SARS-CoV-2 spread based on a compartmental model scheme (Fig. 1), and the model was interpreted from simulating the evolution of COVID-19 epidemiology^29,30^. In our study, the near-real-time reproduction number $\Re_{{\text{t}}}$ represents the time-varying number of secondary cases generated by per infected individual. The transmissibility in baseline scenario is estimated according to the number of contacts within and outside of the household combining with secondary attack rate^31^. The $\Re_{{\text{t}}}$ value introduced in our model of the baseline scenario could well-match the corresponding $\Re_{{\text{t}}}$ estimated based on the real-world surveillance data (https://covid19.who.int/). $\Re_{{\text{t}}}$ was estimated by R package EpiEstim^32^, and the mean serial interval and standard deviation were referred to a previous study^33^. The simulated results were fitted well with the confirmed epidemiological data reported by health authorities during the simulation period (Fig. S1).

Susceptibility to SARS-CoV-2 infection was assumed to be heterogeneous according to the doses administered and experienced SARS-CoV-2 infection. Vaccine-induced protection and natural protection after recovery from the infection are assumed to partially reduce susceptibility to SARS-CoV-2 Omicron infection. As vaccination has plateaued and the number of newly vaccinated individuals could be negligible, the coverages of the subpopulations vaccinated with respective dose are set to constant. Although neutralizing antibody levels increased exponentially after booster dose, immune protection is assumed to wane^34,35^. After waning of protection, individuals are considered fully susceptible, and Omicron can substantially escape from vaccine neutralization^36^. Therefore, the partial vaccination confers no protection, and the effectiveness of full vaccination is assumed at 10% given the six-month recession period (based on UK Health Security Agency COVID-19 vaccine surveillance report: Week 10; 2022). Recovered individuals are 90.7% less likely to develop Omicron reinfection upon an infectious contact (based on UK Health Security Agency COVID-19 vaccine surveillance report: Week 10; 2022). A sensitivity analysis is performed using a vaccine effectiveness of 80.5%^37^ and 94.0%^38^, respectively.

We simulated COVID-19 disease burden in respect to asymptomatic carriers, actively infected cases, hospitalization, severely symptomatic cases, recovered cases and deaths in different scenarios in the presence/absence of molnupiravir treatment. Asymptomatic carriers can spontaneously recover without proceeding to active infection with clear symptoms, which is equivalent to asymptomatic infection. Asymptomatic and symptomatic individuals were assumed to be equally infectious^39^. Hospitalization was regarded as quarantine, whereas the infected but nonhospitalized individuals largely contributed to disease transmission. Approximately one quarter of hospitalized patients experience severe symptoms requiring intensive care in the baseline. Although individuals receiving different types or doses of vaccine are non-homogeneous in the subpopulations of asymptomatic carries, patient, hospitalizations, severely affected, and mortality compartments, the mix of vaccination structures through our simulation duration is assumed to be stable, and the composite transfer rates across compartments have been adjusted for vaccination status.

All parameters of the model were derived from the studies on Omicron-dominant wave or reported data by authorities. The duration between onset to recovery in nonhospitalized patients was 8.6 days in the absence of molnupiravir treatment^40,41^. A proportion of actively infected cases require hospitalization, delayed by an average time of 4 days between symptom onset and hospitalization^42^. Survivors were discharged from hospital after an average of 6 days of admission, and non-survivors were delayed by an average time of 5 days between admission and death^43^. In general, all the severe patients are assumed to be able to admit to hospital, and no fatality could be developed from the non-severe cases. Heterogeneous parameters, including transmission rate, vaccination coverage, and proportion of hospitalization were stratified based on countries in line with real-world data. Our model is initialized according to the observed data reported by Our World in Data as of 01 April, 2022 and run considering daily time steps (ourworldindata.org/coronavirus-data-explorer). Further details are described in Supplementary Information. Although there is no specific guidelines for mathematical modeling study, this study was performed in compliance with some guidelines of STROBE, when applicable.

### Scenarios setting

To explore the impact of molnupiravir treatment on containing COIVD-19 spread, we ran a set of simulations in which no molnupiravir treatment is implemented and the social activity level follows the degree in Week 12, 2022 as a baseline scenario (effective $\Re_{{\text{t}}}$ is 0.83, 0.88, 0.92 in Denmark, UK, and Germany at the beginning of simulations). We compared it with scenarios where molnupiravir treatment for total infections or only symptomatic patients. For insight into the burden of resuming pre-pandemic social activity level, we estimated the transmission rate under hierarchical degrees of social activity patterns which were reflected in the daily number of human social contacts. In the scenario of complete resumption of pre-pandemic social activities, molnupiravir treatment is implemented for symptomatic cases to assess the cost of living with virus. Considering the availability of molnupiravir prescription, scenarios setting hierarchical timeliness and hierarchical coverage of treatment after symptom onset were simulated. In the scenarios of molnupiravir treatment timeliness, the percentage of viral RNA positive dynamics by days was referred to a report from National Institute of Infectious Diseases Disease Control and Prevention Center, Japan (https://www.niid.go.jp/niid/en/2019-ncov-e/10884-covid19-66-en.html). Sensitivity analyses were performed on the heterogeneous reinfection possibility, the infectiousness of Omicron BA.1, BA.2 and XE with incremental secondary attack rate, and heterogeneous vaccine protection efficacy.

### Implementation of molnupiravir treatment

Clinical trials have reported that molnupiravir treatment significantly accelerated SARS-CoV-2 infectious virus elimination and numerically reduced death rate of COVID-19 patients^6,7^. Implementing molnupiravir treatment in nonhospitalized cases was postulated to reducing contagious, shortening infection duration, and preventing adverse outcome. More specifically, based on the phase 2a clinical trial, 800 mg molnupiravir treatment had an average infectious virus positive rate of 13.4% throughout 5 days after onset as compared with 25.0% in those who received placebo^7^. Thus, the transmission rate was assumed to be reduced by 46.4% with molnupiravir. The median clearance time of viral RNA was accelerated with 800 mg molnupiravir treatment compared with placebo groups: 27 days versus 14 days^7^. Considering this efficacy, the recovery time of nonhospitalized infection was set to be reduced by 48.1% after molnupiravir treatment. In the phase 2/3 trial of molnupiravir, 4.1% of participants at increased risk for severe illness required further therapy in the molnupiravir group versus 7.4% of those in the placebo group (https://doi.org/10.1056/EVIDoa2100043), which was assumed as 44.6% reduction of the fraction of hospitalization developing into severe illness with molnupiravir treatment. A phase 3 trial of treating nonhospitalized COVID-19 cases evaluated the rates of hospitalization and death through 29 days follow-up. There was 6.3% of patients requiring hospitalization in the molnupiravir group and 9.2% in the placebo group, and 0.1% death rate was reported in the molnupiravir group while 1.3% in the placebo group^6^. Thus, the fraction of hospitalization and fatality rate were lowered by 31.5% and 89% with molnupiravir in simulation, respectively. We incorporated these key effects into our models to assess the impact of molnupiravir treatment in different subpopulations with different implementation strategies.

## Supplementary Information

Supplementary Information.

## Data Availability

All data needed to evaluate the conclusions in the paper are present in the paper and/or the Supplementary Information.
