# Supplementary material for: Treating SARS-CoV-2 Omicron variant infection by molnupiravir for pandemic mitigation and living with the virus: a mathematical modeling study
Source: Sci Rep. 2023 Apr 4;13:5474. doi: 10.1038/s41598-023-32619-z (PMC10071263; doi:10.1038/s41598-023-32619-z)
Supplement: Supplementary file 1 — Supplementary Information. [file 41598_2023_32619_MOESM1_ESM.pdf]

Supplementary Information

Treating SARS-CoV-2 Omicron variant infection by molnupiravir for pandemic mitigation and living with the virus: a mathematical modeling study

Model Formulation

The mathematical model describes the evolution of eleven subpopulations: susceptible and not vaccinated or partly initial vaccinated (S), fully initial vaccinated (V_2), booster dose vaccinated (V_3), latent (L), asymptomatic carriers (A), actively infected but nonhospitalized (I), hospitalized (H), severely symptomatic (C), recovered (R) and dead (D) in different stages overtime. This SEIR-based epidemiological model was adopted from recent studies of modeling epidemics in Italy (Giordano et al., 2020, Gatto et al., 2020a, Truelove et al., 2020), and the basic characteristics were described as follows.

Susceptible individuals (S, V_2, V_3) experiencing homogeneous susceptibility to SARS-CoV-2 infection reduced by incremental vaccine doses. Latently infected individuals (L) have been infected but without symptom and not infectious. Asymptomatic carriers (A) have no symptoms and will eventually develop into symptomatic infections but have the ability to infect susceptible individuals (Gandhi et al., 2020). They are non-life-threatening with a similar transmissibility when comparing with the symptomatic ones. The actively infected but nonhospitalized cases (I) are ailing with symptoms. The hospitalized COVID-19 patients (H) usually have clear symptoms, but they are quarantined with low risk of transmitting the virus. The severely symptomatic patients (C) generally requiring intensive care with a higher mortality rate (Fig. 8). The compartmental model system consists of following ten equations:

$$
\begin{aligned}
\dot{S} &= -\beta(L+A+I)\frac{S}{N} \\
\dot{V}_2 &= -\beta(1-\upsilon_2)(L+A+I)\frac{V_2}{N} \\
\dot{V}_3 &= -\beta(1-\upsilon_3(t))(L+A+I)\frac{V_3}{N} \\
\dot{L} &= (\beta S+\beta(1-\upsilon_2)V_2+\beta(1-\upsilon_3(t))V_3+\beta(1-\mu)R)\frac{(L+A+I)}{N}-\alpha L \\
\dot{A} &= (1\text{-}b)\,\alpha L-\gamma_A A \\
\dot{I} &= b\alpha L-hI-\gamma_I I \\
\dot{H} &= hI-\gamma_H H-\sigma H \\
\dot{C} &= \sigma H-\gamma_H C-dC \\
\dot{R} &= \gamma_H C+\gamma_H H+\gamma_I I+\gamma_A A-\beta(1-\mu)R\frac{(L+A+I)}{N} \\
\dot{D} &= dC
\end{aligned} \tag{1}
$$

The key parameters are mainly derived from studies of modeling epidemics and epidemiological characterization of Omicron cases (See details in Table S1). The reproduction number $\Re_0$ of our

simulation model in line with transmission rate is stratified by contacts within and outside of the household: $\Re_0 = SAR_h N_h + SAR_c N_c$, where SAR_h and SAR_c are the secondary attack rates within household and outside household, N_h and N_c are the numbers of at-risk contacts exposed within household and outside household, respectively (Liu et al., 2020). Generally, $\Re_0 = \beta T$, where T is the infection period of an infected one(Wu et al., 2020), so $\beta = SAR_h n_h + SAR_c n_c$ in which n_h and n_c are the daily numbers of at-risk contacts. The secondary attack rates and contact patterns were collected in the investigation by UK Health Security Agency (UKHSA) and adjusted for the SARS-CoV-2 Omicron variants mix (BA.1 and BA.2) status as follows (UKHSA, 2022c, UKHSA, 2022d).

$$\beta = \begin{bmatrix} P_{BA.1} & P_{BA.2} \end{bmatrix} \begin{bmatrix} SAR_h^{BA.1} & SAR_c^{BA.1} \\ SAR_h^{BA.2} & SAR_c^{BA.2} \end{bmatrix} \begin{bmatrix} n_h \\ n_c \end{bmatrix} \tag{2}$$

$P_{BA.1}$ and $P_{BA.2}$ represent the shares of SARS-CoV-2 Omicron variant BA.1 and BA.2 in all analyzed sequences, respectively. The secondary attack rates within household and outside household $SAR_h^{BA.1}$ and $SAR_c^{BA.1}$ of Omicron variant BA.1 are differentiated from those of BA.2.

And the numbers of susceptible contacts n_h and n_c are reflected by different social activity level, which are detailed in Table S2. The number of human social contacts in pandemic social activities scenario was captured from weekly statistics for National Health Security (NHS) Test and Trace, in which the infection data was from 27 January and 09 February 2022, and contact tracing data was between 10 February and 23 February 2022 by UKHSA (2022d). The according reproduction number in the beginning of simulation was around 0.9; The number of social contacts in the scenario of partially returning to pre-pandemic social activities referred to the technical briefing reported by UKHSA, where case test dated 1 January to 14 February 2022 and contact tracing data as of 8 March 2022 (UKHSA, 2022c). The according reproduction number in the beginning of simulation was around 1.5; The number of social contacts in scenario of resuming pre-pandemic social activities was obtained from social contact matrix in pre-pandemic investigation (Del Valle et al., 2007).

The VE of partly initial vaccine is negligible. The VE of fully initial vaccine is assumed at 10% (UKHSA, 2022a). The VE of booster vaccine dose is assumed waning from 0.49 against Omicron BA.1 and from 0.46

against Omicron BA.2. The VE was adjusted for the SARS-CoV-2 Omicron variants mix status in the simulated model of Denmark, the United Kingdom. Germany.

The transfer rates across compartmental subpopulation I, H, C, R, and D were estimated considering the average delay time. The convert rate of infections with clear symptoms admitted to hospitals h is calculated through real-world proportion of hospitalizations in symptomatic infections dividing the average delay time of hospital admissions. The convert rate of infections with mild/moderate symptoms to severe diseases σ could be obtained by the real-world proportion of severely symptomatic infections in patients and in-hospital time. The recovery rate of asymptomatic infected individuals γ_A is set as the inverse of median recovery time of non-hospitalized cases. The recovery rate of infected individuals with clear symptoms but not hospitalized γ_I is acquired by the reported proportion of non-hospitalized cases in patients dividing the median recovery time of non-hospitalized cases. The recovery rate of hospitalized patients γ_H is calculated through real-world percentage of rehabilitation in hospitalizations and the median in-hospital time. The fatality rate of infected individuals with severe symptoms d is obtained from the proportion of deaths in hospitalizations and median in-hospital time of individuals who finally died.

The median incubation period was observed approximately as 3 days (Jansen et al., 2021). The mean infection episode duration was 11.6 days (Cohen et al., 2021). The recovery period of asymptomatic or actively infected cases without being admitted to hospitals is assumed as 8.6 (11.6 minus 3) days. Hospital admissions delayed by an average time of 4.0 days (Gatto et al., 2020b). The median time from admission to an in-hospital outcome was 5 days for individuals who died, and 6 days for individuals who were discharged alive (Wolter et al., 2022b). The fraction of symptomatic patients admitted to hospitals and the proportion of infections with mild/moderate symptoms developed to severe diseases in Denmark and the United Kingdom were calculated referring to the epidemiological characterization observed by (Fonager et al., 2022, Wolter et al., 2022a) and adjusting for SARS-CoV-2 Omicron variants mix (BA.1 and BA.2) status, while those of Germany were estimated according to reported data (Data, 2022).

Estimating time-varying reproduction number

The time-varying reproduction number of $\Re_t$ was estimated on account of incidences of daily cases. Based on the time-dependent method proposed by Wallinga et al. (2004) and Obadia et al. (2012), reproduction number $\Re_t$ could be computed by averaging over all transmission networks compatible with incidences, and the generation time distribution of incident cases over consecutive time units is ω. The likelihood p_{ij} that case i with confirmation at time t_i infected by case j with confirmation at time t_j is presented as:

$$p_{ij} = \frac{N_i\omega(t_i - t_j)}{\sum_{i \neq k} N_i\omega(t_i - t_k)}$$

The effective reproduction number for case j is the sum over all cases i, weighted by the relative likelihood that case i has been infected by case j, $\Re_j = \sum_i p_{ij}$. All cases with the same date of conformation is averaged as $\Re_t = \frac{1}{N_t}\sum_{\{t_j = t\}} \Re_j$, which is the reproduction number at time t.

We estimated $\Re_t$ it by R package EpiEstim based on mentioned equation derivations (Cori et al., 2013), where the mean serial interval is 3.0 days and its standard deviation is 2.9 (Song et al., 2022).

Sensitivity analysis on parameters

If we only considered the compartment L, A, I, H, and C. $S^{tot} = S + (1-\upsilon_2)V_2 + (1-\upsilon_3(t))V_3 + (1-\mu)R$.

$$\mathrm{F} = \begin{pmatrix} \beta\frac{S^{tot}}{N}(L + A + I) \\ 0 \\ 0 \\ 0 \\ 0 \end{pmatrix}, \quad \mathrm{V} = \begin{pmatrix} \alpha L \\ -\alpha(1-b)L + \gamma_A A \\ -b\alpha L + hI + \gamma_I I \\ -hI + \gamma_H H + \sigma H \\ -\sigma H + \gamma_H C + dC \end{pmatrix}$$

F represents the rate of emergence of new infected individuals, V represents the rate of interconversion between compartments. Calculate the derivatives of F and V with respect to

$x=\left(L,A,I,H,C\right)^T$ respectively, and then substitute the initial data $\left(S_0^{tot'},L_0',A_0',I_0',H_0',C_0',R_0'\right)$, we can obtain Jacobian matrices:

$$F=\frac{\partial \mathrm{F}}{\partial x}\begin{pmatrix} \beta\frac{S_0^{tot}}{N} & \beta\frac{S_0^{tot}}{N} & \beta\frac{S_0^{tot}}{N} & 0 & 0 \\ 0 & 0 & 0 & 0 & 0 \\ 0 & 0 & 0 & 0 & 0 \\ 0 & 0 & 0 & 0 & 0 \\ 0 & 0 & 0 & 0 & 0 \end{pmatrix} \quad V=\frac{\partial \mathrm{V}}{\partial x}\begin{pmatrix} \alpha & 0 & 0 & 0 & 0 \\ -\alpha(1-b) & \gamma_A & 0 & 0 & 0 \\ -\alpha b & 0 & h+\gamma_I & 0 & 0 \\ 0 & 0 & -h & \gamma_H+\sigma & 0 \\ 0 & 0 & 0 & -\sigma & \gamma_H+d \end{pmatrix}$$

The reproduction number $\Re_t$ is then the spectral radius (i.e., the largest modulus of the eigenvalues) of the next-generation matrix FV^{-1}, as shown:

$$FV^{-1}=\begin{pmatrix} \frac{\beta S_0^{tot}}{\alpha \mathrm{N}}-\frac{\beta(b-1)S_0^{tot}}{\gamma_A \mathrm{N}}+\frac{\beta b S_0^{tot}}{(h+\gamma_I)\mathrm{N}} & \frac{\beta S_0^{tot}}{\gamma_A \mathrm{N}} & \frac{\beta S_0^{tot}}{(h+\gamma_I)\mathrm{N}} & 0 & 0 \\ 0 & 0 & 0 & 0 & 0 \\ 0 & 0 & 0 & 0 & 0 \\ 0 & 0 & 0 & 0 & 0 \\ 0 & 0 & 0 & 0 & 0 \end{pmatrix}$$

$$\begin{aligned}\Re_t=\rho\left(FV^{-1}\right)&=\frac{S_0^{tot}}{N}-\left(\frac{\beta}{\alpha}-\frac{\beta(b-1)}{\gamma_A}+\frac{\beta b}{h+\gamma_I}\right)\\ &=\frac{S+(1-\upsilon_2)V_2+(1-\upsilon_3(t))V_3+(1-\mu)R}{N}-\left(\frac{\beta}{\alpha}-\frac{\beta(b-1)}{\gamma_A}+\frac{\beta b}{h+\gamma_I}\right)\end{aligned}$$

The reproduction number $\Re_t$ represents the human-to-human transmission number of secondary cases generated by per infected individual. The dividing line between sustaining transmission and epidemic decline is 1. The parameters of transmission rate β, incubation period $\frac{1}{\alpha}$, the proportion of symptomatic infection b, the fraction of hospitalization h , the recovery rate γ_A and γ_I. v_2, v_3, and μ are the efficacy protected by vaccination and perious infection.

Supplementary Tables

Parameter	Definition	Values Denmark	Values UK	Values Germany	Source
β	transmission rate of infected but unhospitalized	0.140(95%CI 0.133-0.146)	0.131(95%CI 0.125-0.137)	0.126(95%CI 0.121-0.131)	(UKHSA, 2022c)
v_2	vaccine efficacy of fully initial doses		10%		(UKHSA, 2022a)
v_3	vaccine efficacy of booster doses		time-decaying from 49% (95%CI 41%-55%)		(UKHSA, 2022a)
μ	protection efficacy against repeat infection		90.7%		(UKHSA, 2022c)
$1/\alpha$	median incubation time		3 days		(Li et al., 2020)
b	proportion of symptomatic infections		65.0%		(Espenhain et al., 2021)
h	convert rate of infections with clear symptoms admitted to hospitals	0.0115	0.0115	0.002	(Gatto et al., 2020b, Fonager et al., 2022)
σ	convert rate of infections with mild/moderate symptoms to severe diseases	0.035	0.035	0.045	(Espenhain et al., 2021)
$1/\gamma_A$	median recovery time of asymptomatic infected individuals		8.6 days		(Cohen et al., 2021, Jansen et al., 2021)
γ_I	recovery rate of infected individuals with clear symptoms but not hospitalized		11.3%		(Fonager et al., 2022, Cohen et al., 2021, Jansen et al., 2021)
γ_H	recovery rate of hospitalized patients including severely		15.0%		(Wolter et al., 2022b)

	symptomatic ones				
d	fatality rate of infected individuals with severe symptoms	0.095	0.095	0.098	(Wolter et al., 2022b, Fonager et al., 2022)

Table S1. Key parameters of the mathematical model in the baseline scenario.

Parameter	Definition	Values			Source
		Denmark	UK	Germany	
$P_{BA.1}$	share of SARS-CoV-2 Omicron variant BA.1	98.8%	93.7%	72.0%	(UKHSA, 2022c, Consortium, 2022, Institue, 2022)
$P_{BA.2}$	share of SARS-CoV-2 Omicron variant BA.2	1.2%	6.3%	28.0%	
		BA.1	BA.2	XE	
SAR_h	secondary attack rate within household	10.7% (95% CI 10.6%-10.8%)	13.6% (95% CI 13.2%-14.0%)	1.1 times of BA.2	(UKHSA, 2022c)
SAR_c	secondary attack rate outside household	4.2% (95% CI 4.0%-4.3%)	5.3% (95% CI 4.7%-5.8%)	1.1 times of BA.2	
		Pandemic	Partly return to pre-pandemic	Pre-pandemic	(UKHSA, 2022c, UKHSA, 2022d, Del Valle et al., 2007)
n_h	number of at-risk contacts exposed within household	0.55	2.07	2.00	
n_c	number of at-risk contacts exposed outside household	0.37	2.46	6.66	

Table S2. Parameters related to transmission rate estimation.

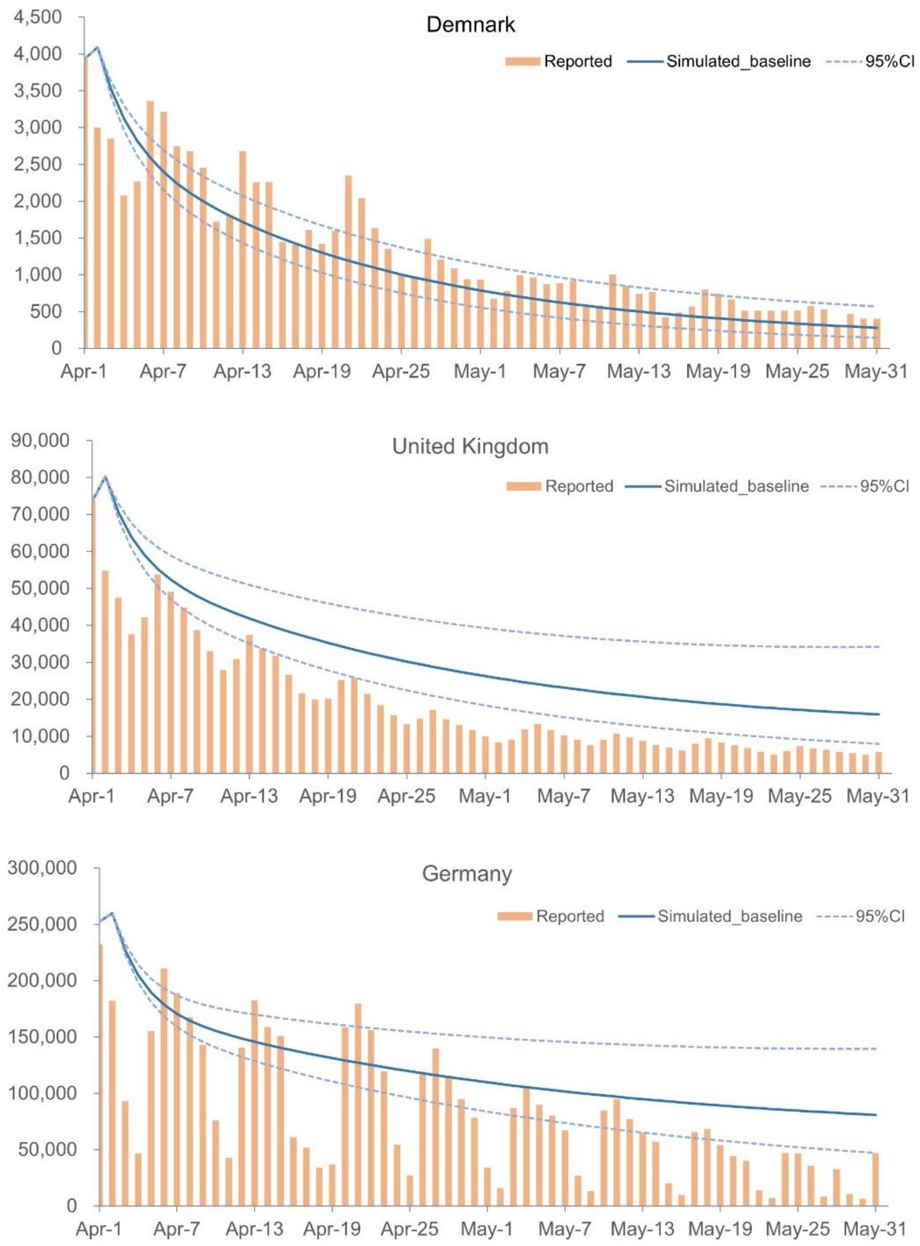

Fig. S1. Comparing the simulated infections in the baseline scenario and real-world reported data in the simulation period.

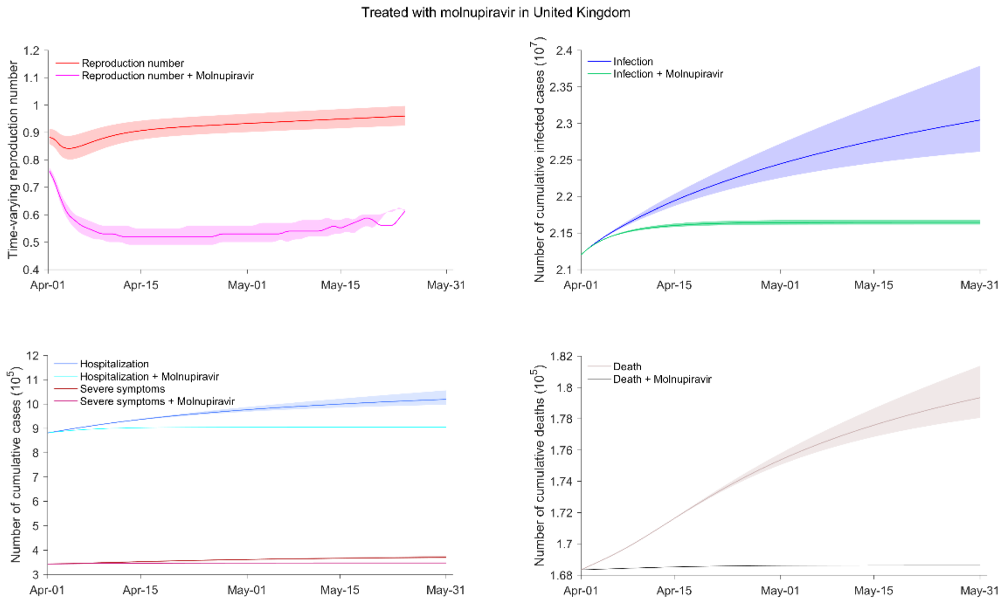

Fig. S2. Impact of molnupiravir treatment for all nonhospitalized cases on mitigating COVID-19 pandemic in UK. This is related to Figure 4 and see more details in the legend of Figure 4.

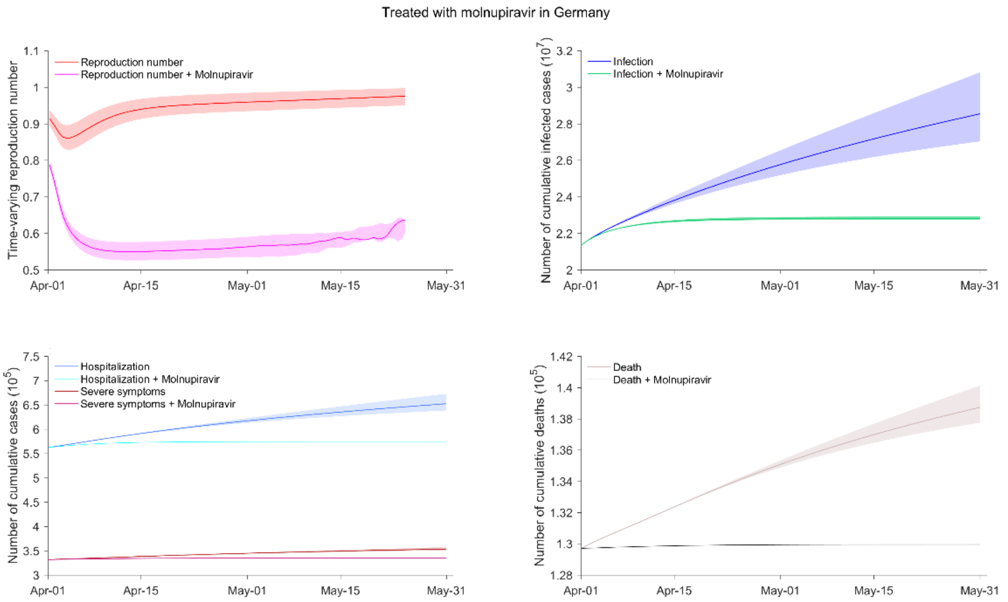

Fig. S3. Impact of molnupiravir treatment for all nonhospitalized cases on mitigating COVID-19 pandemic in Germany. This is related to Figure 4 and see more details in the legend of Figure 4.

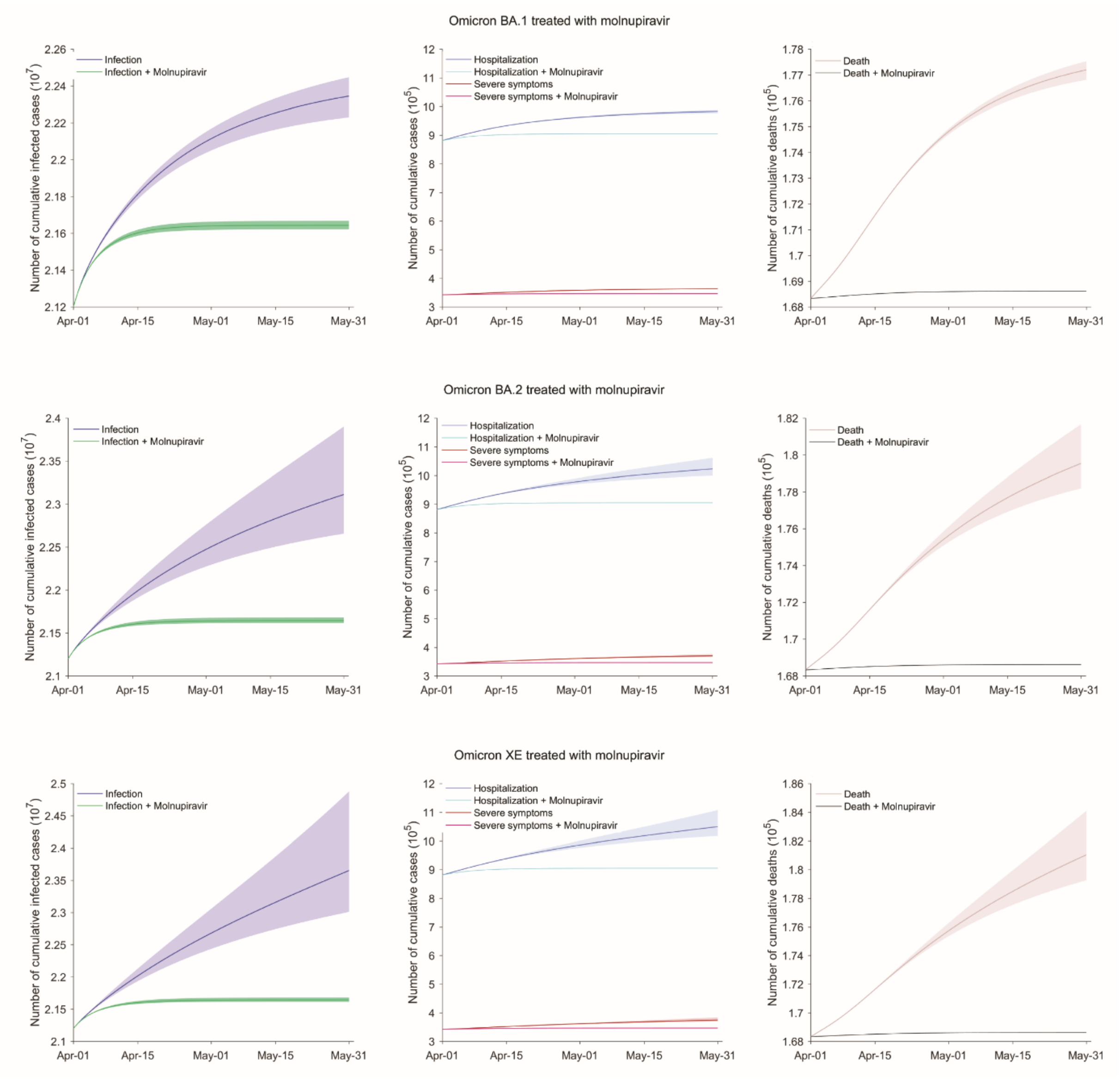

Fig. S4. Comparing the impact of molnupiravir treatment on different lineages of SARS-CoV-2 Omicron variant in UK. (a) Omicron BA.1. **(b)** Omicron BA.2. **(c)** Omicron XE. The vaccine effectiveness against Omicron BA.2 and XE is lower than that against Omicron BA.1 (UKHSA, 2022a). The transmission rate of BA.2 is approximately 30% higher than that of BA.1 (UKHSA, 2022c). Omicron XE is a recombinant of Omicron BA.1 and BA.2., which is about 10% more transmissible than BA.2 sub-variant of Omicron (UKHSA, 2022b, UKHSA, 2022c). There are also differences in hospitalization risk and vaccine efficacy between BA1 and BA2 (UKHSA, 2022a, Fonager et al., 2022). The settings of the social activity level, the reinfection proportion, the treatment implementation time, and the molnupiravir treatment effectiveness for individuals are identical to those in Figure 4. The shadow parts represent the 95% confidence interval which are related to parameters.

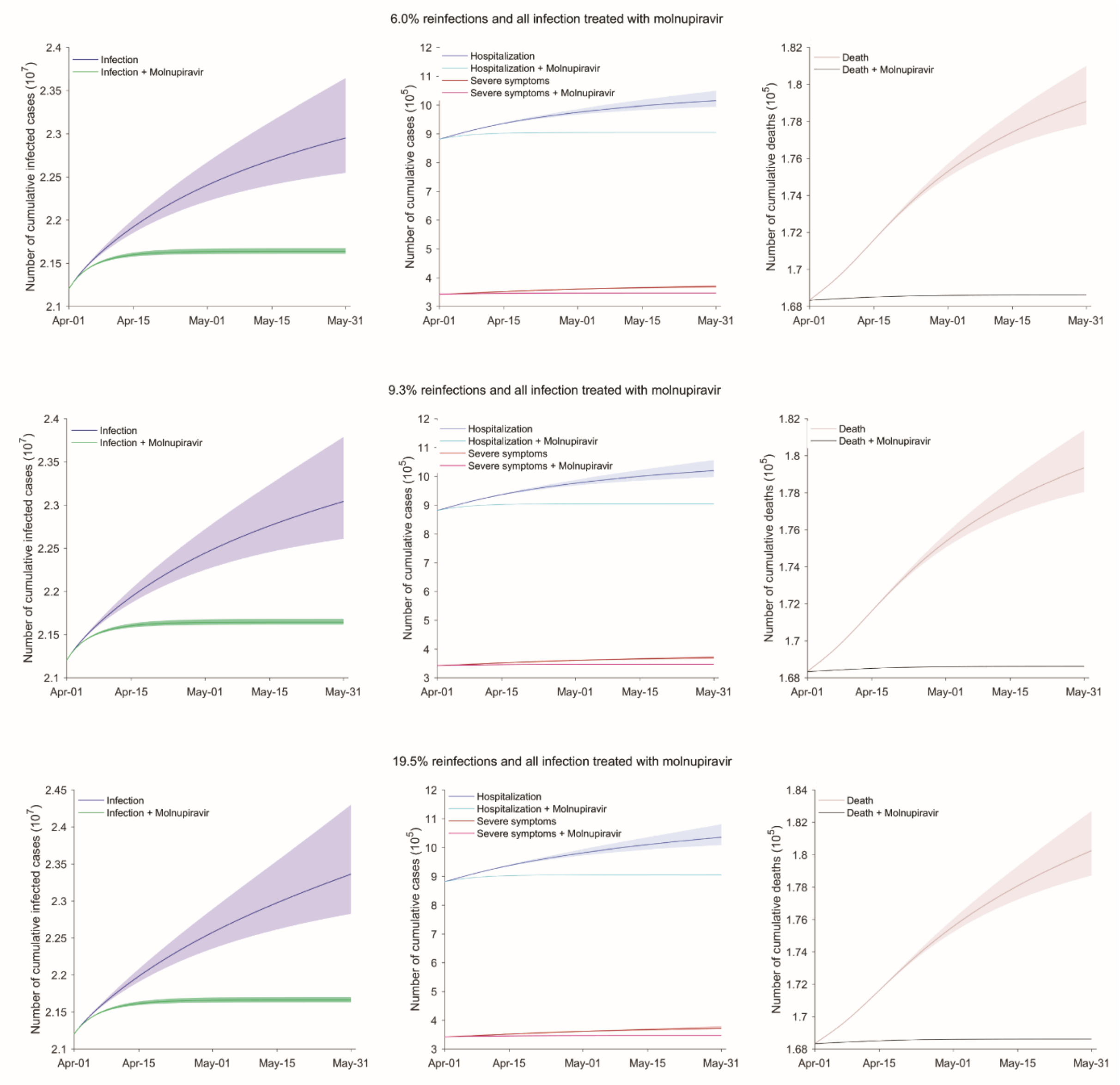

Fig. S5. Sensitivity analysis of molnupiravir treatment assuming different efficacies of protecting reinfection in UK. (a) 94.0% (Fonager et al., 2022). **(b)** 90.7% (UKHSA 2022c). **(c)** 80.5% (Hansen et al., 2021). The settings of the social activity level, the treatment implementation time, dominating pango lineages, and the molnupiravir treatment effectiveness for individuals are identical to those in Figure 4. The shadow parts represent the 95% confidence interval which are related to parameters.

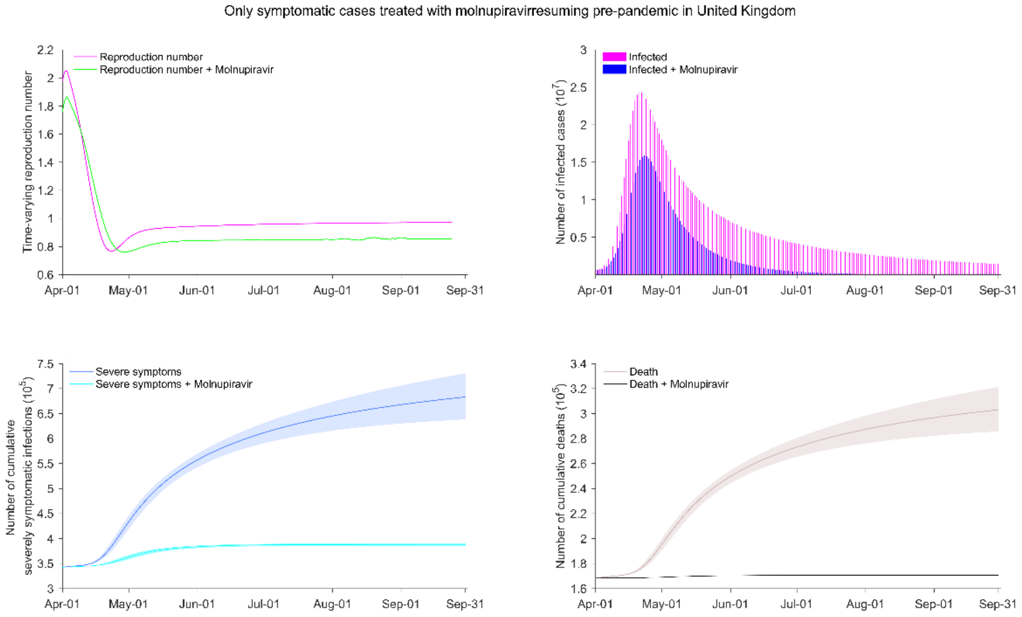

Fig. S6. Impact of molnupiravir treating symptomatic patients on mitigating COVID-19 pandemic resuming pre-pandemic social activities in UK. This is related to Figure 8 and see more details in the legend of Figure 8.

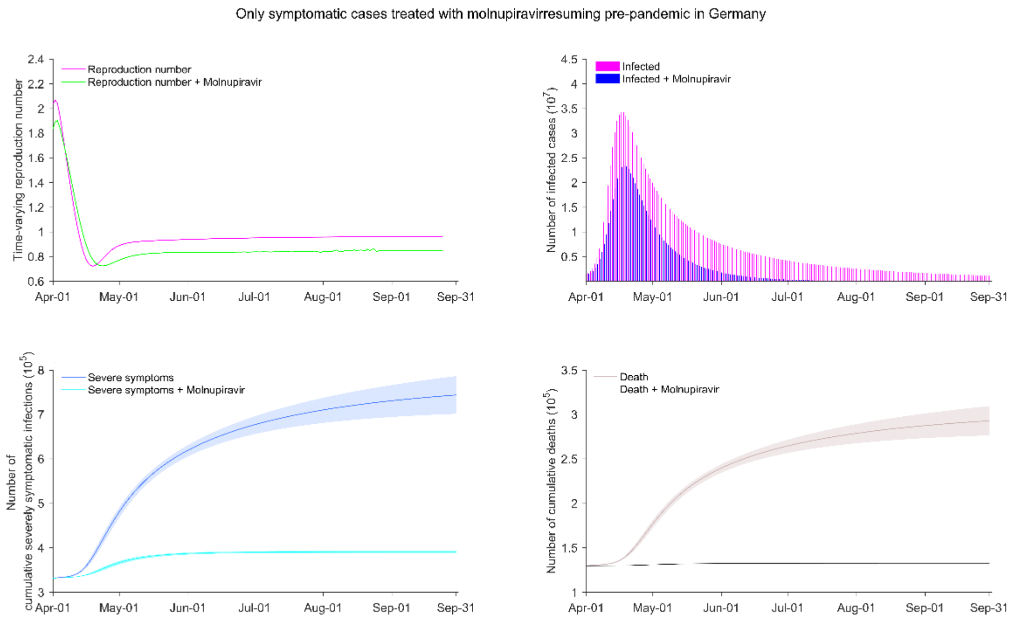

Fig. S7. Impact of molnupiravir treating symptomatic patients on mitigating COVID-19 pandemic resuming pre-pandemic social activities in Germany. This is related to Figure 8 and see more details in the legend of Figure 8.

Reference

1. Cohen C, Kleynhans J, Gottberg Av, et al. SARS-CoV-2 incidence, transmission and reinfection in a rural and an urban setting: results of the PHIRST-C cohort study, South Africa, 2020-2021. Lancet Infectious Disease. 2022; 22(6):821-834. doi: 10.1016/S1473-3099(22)00069-X.
2. Cori A, Ferguson NM, Fraser C, Cauchemez S. A new framework and software to estimate time-varying reproduction numbers during epidemics. Am J Epidemiol. 2013;178(9):1505-12. doi:10.1093/aje/kwt133.
3. Danish Covid-19 Genome Consortium. Genomic overview of SARS-CoV-2 in Denmark. Viruses2022.
4. Del Valle SY, Hyman JM, Hethcote HW, Eubank SG. Mixing patterns between age groups in social networks. Social Networks. 2007;29(4):539-54. doi: 10.1016/j.socnet.2007.04.005.
5. Espenhain L, Funk T, Overvad M, et al. Epidemiological characterisation of the first 785 SARS-CoV-2 Omicron variant cases in Denmark, December 2021. Eurosurveillance. 2021;26(50):2101146. doi: 10.2807/1560-7917.ES.2021.26.50.2101146.
6. Fonager J, Bennedbæk M, Bager P, et al. Molecular epidemiology of the SARS-CoV-2 variant Omicron BA.2 sub-lineage in Denmark, 29 November 2021 to 2 January 2022. Eurosurveillance. 2022;27(10):2200181. doi:10.2807/1560-7917.ES.2022.27.10.2200181.
7. Gandhi M, Yokoe DS, Havlir DV. Asymptomatic Transmission, the Achilles' Heel of Current Strategies to Control Covid-19. N Engl J Med. 2020;382(22):2158-60. doi:10.1056/NEJMe2009758.
8. Gatto M, Bertuzzo E, Mari L, et al. Spread and dynamics of the COVID-19 epidemic in Italy: Effects of emergency containment measures. Proc Natl Acad Sci U S A. 2020;117(19):10484-91. doi:10.1073/pnas.2004978117
9. Gatto M, Bertuzzo E, Mari L, et al. Spread and dynamics of the COVID-19 epidemic in Italy: Effects of emergency containment measures. proceedings of the national academy of sciences of the United States of America. 2020;117(19):10484-91. doi:10.1073/PNAS.2004978117.
10. German Robert Koch Institue. Coronavirus Disease 2019 (COVID-19) Daily Situation Report by the Robert Koch Institute2022 March 3, 2022.
11. Giordano G, Blanchini F, Bruno R, et al. Modelling the COVID-19 epidemic and implementation of population-wide interventions in Italy. Nat Med. 2020;26(6):855-60. doi:10.1038/s41591-020-0883-7
12. Hansen CH, Michlmayr D, Gubbels SM, Mølbak K, Ethelberg S. Assessment of protection against reinfection with SARS-CoV-2 among 4 million PCR-tested individuals in Denmark in 2020: a population-level observational study. The Lancet. 2021;397(10280):1204-12. doi: 10.1016/S0140-6736(21)00575-4.
13. Jansen L, Tegomoh B, Lange K, et al. Investigation of a SARS-CoV-2 B.1.1.529 (Omicron) Variant Cluster - Nebraska, November-December 2021. MMWR. Morbidity and mortality weekly report.

2021;70(5152):1782-4. doi:10.15585/mmwr.mm705152e3.

14. Li Q, Guan X, Wu P, et al. Early Transmission Dynamics in Wuhan, China, of Novel Coronavirus–Infected Pneumonia. New England Journal of Medicine. 2020;382(13):1199-207. doi:10.1056/NEJMoa2001316.
15. Liu Y, Eggo RM, Kucharski AJ. Secondary attack rate and superspreading events for SARS-CoV-2. The Lancet. 2020;395(10227): e47. doi:10.1016/S0140-6736(20)30462-1.
16. Our World in Data. COVID-19 Data Explorer. 2022.
17. Obadia, T., et al. (2012). "The R0 package: a toolbox to estimate reproduction numbers for epidemic outbreaks." BMC Med Inform Decis Mak12:147.
18. Song, J.S.*, et al.* Serial Intervals and Household Transmission of SARS-CoV-2 Omicron Variant, South Korea, 2021. *Emerg Infect Dis* **28**, 756-759 (2022).
19. Truelove SA, Keegan LT, Moss WJ, et al. Clinical and Epidemiological Aspects of Diphtheria: A Systematic Review and Pooled Analysis. Clin Infect Dis. 2020;71(1):89-97. doi:10.1093/cid/ciz808
20. UK Health Security Agency. COVID-19 vaccine surveillance report: Week 10 2022, 10 March, 2022.
21. UK Health Security Agency. COVID-19 variants identified in the UK 2022, 25 March, 2022.
22. UK Health Security Agency. SARS-CoV-2 variants of concern and variants under investigation in England: Technical briefing 38 2022, 11 March, 2022.
23. UK Health Security Agency. Weekly statistics for NHS Test and Trace (England): 3 to 9 March 2022, 17 March, 2022.
24. Wallinga, J., et al. (2004). "Different epidemic curves for severe acute respiratory syndrome reveal similar impacts of control measures." Am J Epidemiol 160 (6):509-516.
25. Wolter N, Jassat W, group DA-Ga, von Gottberg A, Cohen C. Clinical severity of Omicron sub-lineage BA.2 compared to BA.1 in South Africa. medRxiv. 2022:2022.02.17.22271030. doi:10.1101/2022.02.17.22271030.
26. Wolter N, Jassat W, Walaza S, et al. Early assessment of the clinical severity of the SARS-CoV-2 omicron variant in South Africa: a data linkage study. The Lancet. 2022;399(10323):437-46. doi:10.1016/S0140-6736(22)00017-4.
27. Wu JT, Leung K, Leung GM. Nowcasting and forecasting the potential domestic and international spread of the 2019-nCoV outbreak originating in Wuhan, China: a modelling study. The Lancet. 2020;395: 689-97. doi:10.1016/S0140-6736(20)30260-9.
